# A Case of Bone Angiosarcoma

**Published:** 2017-01-12

**Authors:** Mahmood Akhavan Tafti, Najmeh Jafari, Jalil Zare, Mohamad Jalal Jafari

**Affiliations:** 1 *Dept. of Surgery, Shahid Sadooghi Hospital, Shahid Sadooghi University of Medical Sciences, Yazd, Iran*; 2 *Dept. of Pathology, Shahid Sadooghi Hospital, Shahid Sadooghi University of Medical Sciences, Yazd, Iran*

**Keywords:** Angiosarcoma, Bone, Vascular Tumor

## Abstract

Primary angiosarcoma of bone is very rare. It occurs more commonly in middle-age and later life, with a male predominance in the ratio of 2:1. Angiosarcoma of bone has a tendency to involve the long tubular bones, and multifocal involvement is common. Here, we present a case of a 69-yr-old man in Shahid Sadooghi Hospital of Yazd in 2014 that had angiosarcoma of the left tibia. He was treated with curettage and bone fixation. Two months after the surgery, he died of pulmonary metastasis.

## Introduction

Vascular malignancies are one of the rare malignancies in bone that have variable clinicopathologic features. Angiosarcoma is an aggressive malignant vascular tumor. Origin of tumor cells is mesenchymal cells. This tumor occurs in different sites, but intraosseous variation is rare (1). The most common bones involved are femur and tibia. It occurs in young adults and in men more than women (2).

These tumors have a high recurrence. The risk of lymph node involvement and distant metastases is high. Patients with this aggressive cancer usually die within one year after of diagnosis (3). Bone epithelioid angiosarcoma is misdiagnosed as a metastatic carcinoma, due to the presence of epithelioid cells. Patients should be evaluated precisely regarding clinical and histopatological characteristics for better treatment and higher prognosis (4). 

This article reports a case of angiosarcoma of tibia in a 69-yr-old man. The tumor was diagnosed after radiographic examination and biopsy, although, immunohistochemical tests were performed to confirm the diagnosis and rule out other lesions.

## Case report

**Fig. 1:**
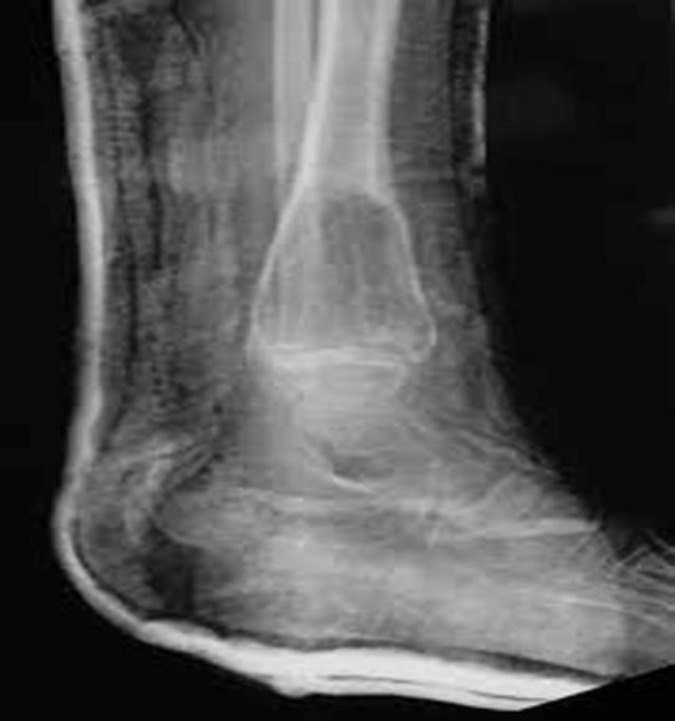
A bone lytic lesion with undefined margins in the middle part of tibia

The presented case was a of 69-yr-old man in Shahid Sadooghi Hospital, Yazd, Iran 2014 who had undergone brain tumor surgery and after one year was readmitted to the hospital due to severe pain in the legs and ankles. Foot pain was so severe that the patient was unable to walk. In clinical examination, anterior tibial and femoral areas and pelvis were tender and sore to touch. Eminence was observed in 1/3 left tibia distal. The patient had no history of trauma. Plain radiographs showed a bone lytic lesion with undefined margins in the inferior part of tibia (Fig. 1). 

**Fig. 2:**
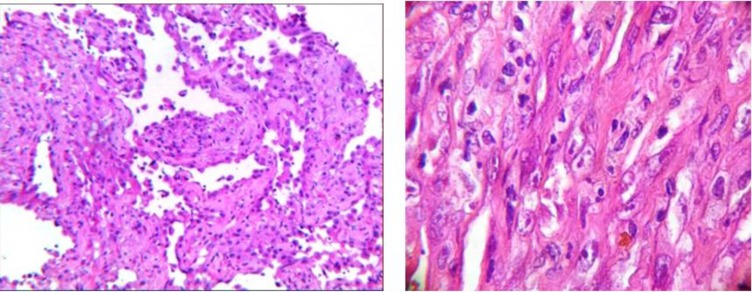
An (x100) &B (x400): many spaces lined by epithelioid cells with vesicular nuclear and small central nucleoli and low mitotic activity supported by fibrous stroma

In biopsy taken from the patient, several pieces of soft tissue with a cream-brown color, similar to cyst wall-sized 3x3x2 cm were observed. Histopathologically, sections show multiple fragments of a tumoral tissue composed of many spaces lined by epithelioid cells with vesicular nuclear and small central nucleoli and low mitotic activity supported by fibrous stroma with multifocal hemosiderin deposition (Fig. 2). 

**Fig 3 (A & B).**
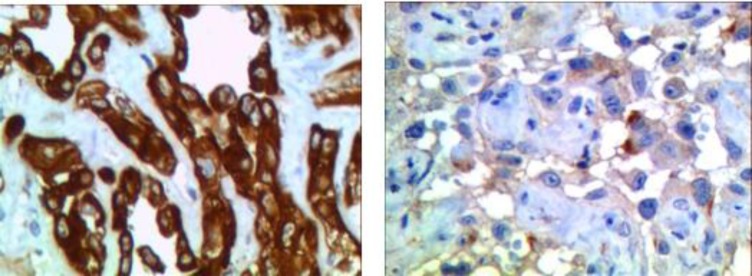
Shows thin-walled vessels within the marrow spaces of cancellous bone with associated reactive fibrosis (H&E stain X40

Immunohistochemical testing for VWF, CKAE1/3, EMA was requested to confirm the diagnosis, and rule out other lesions. All markers were positive (Fig. 3). 

**Fig. 4:**
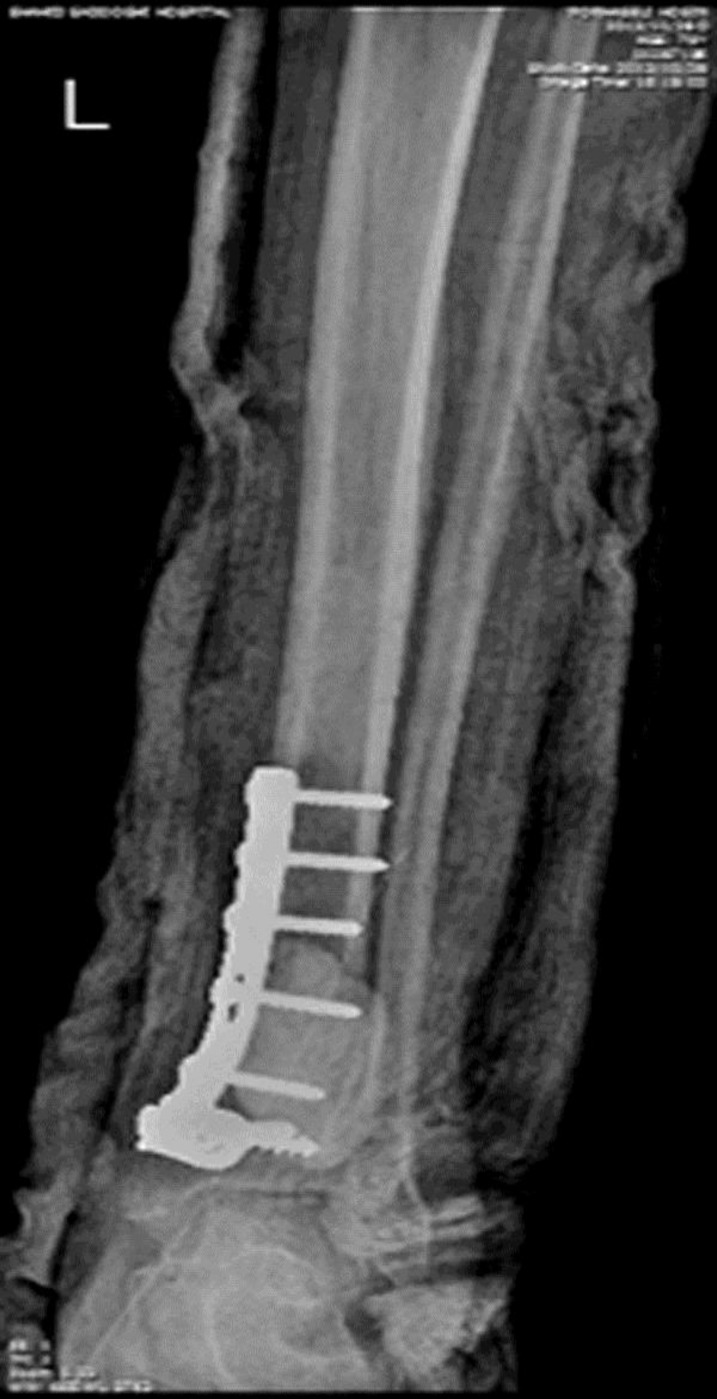
Curettage and bone fixation

In bone marrow biopsy and peripheral blood smears, no evidence of malignancy was found. CT of the abdomen and pelvis was normal. In CT of lung, small opacities were observed in the anterior of right lung upper lobe. Radiography, histopathology, and immunohistochemistry findings confirmed the diagnosis of an intraosseous angiosarcoma. The patient underwent curettage and bone fixation (Fig. 4). After a two-month follow-up, the patient died of pulmonary metastasis.

## Discussion

Angiosarcoma is a malignant vascular neoplasm that commonly occurs in soft tissue but intraosseous angiosarcoma is rare (1). It occurs commonly in older adults and the males are slightly more affected than females (2). Angiosarcoma of bone occurs in tubular bones of the extremities such as femur, tibia, and humerus (5). In our case, clinical characteristics such as age, sex, and site were in accordance with this lesion. Unifocal or multifocal osseous disease may be seen in patients with angiosarcoma. Pain is the most common symptom, however size and localization are important as well (1).

Radiographic appearance of angiosarcoma is variable. This tumor is mostly as a purely lytic mass (5). Because of rapid and destructive growth of this tumor, periosteal reaction may occur. Expansion, cortical permeation and soft tissue mass may occur in intraosseous angiosarcoma (3).

Grossly, angiosarcoma is a bloody and firm tissue with irregular margins. Pathologically, angiosarcoma contains vascular spaces lined with malignant endothelial cells. In some lesions, malignant cuboidal cells with eosinophil cytoplasm and vesicular nuclei are seen. These lesions with an epithelioid appearance are called “epithelioid angiosarcoma”.

Cellularity in high-grade lesions is more than low-grade ones. Epithelioid angiosarcomas are always high-grade (1). Our case had an epithelioid angiosarcoma.

The most bone epithelioid angiosarcomas are immune positive for epithelial and endothelial markers and factor VIII–associated antigen. Among these markers, CD31 has the highest specificity and sensitivity. CD31 and CD34 are expressed in more than 90% of angiosarcomas but specificity of CD34 is poor (4).

In our case, EMA, CKAE1/3, and VWF had positive results, which helped for accurate diagnosis along with clinical, radiographic, and histopathological features.

A case of a 62-yr-old man was reported with angiosarcoma of left tibia. The tumor infiltrated the surrounding soft tissue. CD34 had positive result and histopathological finding was suggestive angiosarcoma. Clinical, radiographic and histopathological finding between our case and this case were similar except that aforementioned case received chemotherapy after surgery. Finally, both cases died due to lung metastasis (6).

A patient was reported with more gauche disease, who had advancing pain in the right knee and femur. After a pathological fracture in leg, primary bone angiosarcoma was evident. Despite similarities in symptoms between our case and the aforementioned one, our case had no systemic disease (7).

An epithelioid angiosarcoma of the left heel was reported in a 64-yr-old woman. In this case, the diagnosis was confirmed by factor 8 and vimentin and CD31. Despite the differences in gender and location in comparison with our case, both died due to lung metastasis (4).

Osteogenic sarcoma- angiomatoid variant is a major differential diagnosis. This lytic mass lesion occurs in long bones. Microscopically, it contains dilated, blood-filled vascular spaces lined by malignant osteoblasts and multinucleated giant cells, and tumor osteoid.

Other differential diagnoses include metastatic carcinoma, hemangioma, and renal cell carcinoma. Vascular markers are useful in differentiation of angiosarcoma in these lesions. Another differential diagnosis is bacillary angiomatosis. In this tumor, neutrophills and clumps of bacteria that stain with the Warthin-Starry silver stain confirms the diagnosis of bacillary angiomatosis (8).

Differentiation of metastatic carcinoma and angiosarcoma is difficult due to multiple bones involvement, occurring in older individuals and present of epithelioid cells. Malignant cells differentiate angiosarcoma from hemangioma (6).

Treatment of these tumors is controversial. Basic treatment is en block resection and adjuvant radiotherapy. The role of chemotherapy is unknown (3). Doxorubicin-based chemotherapy regimens have also been used for angiosarcomas. Patients were treated with doxorubicin-based regimens (9). Local recurrence, early metastases and poor prognosis are indicating that the tumor is aggressive (3).

As in the present case, angiosarcoma had metastasized to the lungs and the patient died after two months.

The combination of surgery and radiotherapy and chemotherapy improves the prognosis (4).

## Conclusion

Bone epithelioid angiosarcoma is a rare tumor and should be meticulously distinguished from vascular tumor and metastatic carcinoma of the bone. This article may provide a better understanding of the pathology of bone epithelioid angiosarcoma and valuable insights for accurate diagnosis, treatment, and prognosis for patients with this deceptive disease.

## Conflict of Interests

The authors declare that there is no Conflict of Interests. 

## References

[1] Baliaka A, Balis GC, Michalopoulou-Manoloutsiou E, Papanikolaou A, Nikolaidou A (2013). primary angiosarcoma of bone. Hippokratia.

[2] Yener S, Yusuf Y, Hakan A, Kerem B (2007). Primary angiosarcoma of the fibula: A case report. Acta Orthop Belg.

[3] Yamashita H, Endo K, Teshima R (2012). Angiosarcoma of the proximal humerus: a case report and review of the literature. J Med Case Rep.

[4] Zhengming Y, Huimin T, Zhaoming Y, Disheng Y (2012). Multicentric epithelioid angiosarcoma of bone. Orthop J.

[5] Unni KK, Inwards CY, Bridge JA, Kindblom L-G, Wold LE (2006). Tumors of the Bones and Joints. AFIP atlas of tumor pathology.

[6] Kudva R, Perveen S, Janardhana A (2010). Primary epithelioid angiosarcoma of bone: A case report with immunohistochemical study. Indian J Pathol Microbiol.

[7] Zver S, Bracko M, Andoljsek D (2010). Primary bone angiosarcoma in a patient with Gaucher disease. Int J Hematol.

[8] Mittal S, Goswami C, Kanoria N, Bhattacharya A (2007). Post-irradiation angiosarcoma of bone. J Cancer Res Ther.

[9] Abraham JA, Hornicek FJ, Kaufman AM, Harmon DC, Springfield DS, Raskin KA (2007). Treatment and outcome of 82 patients with Angiosarcoma. Ann Surg Oncol.

